# Dissecting antenatal care inequalities in western Nepal: insights from a community-based cohort study

**DOI:** 10.1186/s12884-023-05841-w

**Published:** 2023-07-17

**Authors:** Vishnu Khanal, Sangita Bista, Shiva Raj Mishra, Andy H. Lee

**Affiliations:** 1 Nepal Development Society, Bharatpur, Chitwan Nepal; 2 Independent Public Health Consultant, Kathmandu, Nepal; 3 School of Population Health, Curtin University, Perth, Australia

**Keywords:** Pregnancy, Maternal health, Low-and Middle-Income Countries, Sustainable development goals

## Abstract

**Background:**

Antenatal care (ANC) ensures continuity of care in maternal and foetal health. Understanding the quality and timing of antenatal care (ANC) is important to further progress maternal health in Nepal. This study aimed to investigate the proportion of and factors associated with, key ANC services in western Nepal.

**Methods:**

Data from a community-based cohort study were utilized to evaluate the major ANC service outcomes: (i) three or less ANC visits (underutilization) (ii) late initiation (≥ 4 months) and (iii) suboptimal ANC (< 8 quality indicators). Mothers were recruited and interviewed within 30 days of childbirth. The outcomes and the factors associated with them were reported using frequency distribution and multiple logistic regressions, respectively.

**Results:**

Only 7.5% of 735 mothers reported not attending any ANC visits. While only a quarter (23.77%) of mothers reported under-utilizing ANC, more than half of the women (55.21%) initiated ANC visits late, and one-third (33.8%) received suboptimal ANC quality. A total of seven factors were associated with the suboptimal ANC. Mothers with lower education attainment, residing in rural areas, and those who received service at home, were more likely to attain three or less ANC visits, late initiation of ANC, and report receiving suboptimal ANC. Furthermore, mothers from poor family backgrounds appeared to initiate ANC late. Mothers from disadvantaged Madhesi communities tended to receive suboptimal ANC.

**Conclusions:**

Despite a high ANC attendance, a significant proportion of mothers had initiated ANC late and received suboptimal care. There is a need to tailor ANC services to better support women from Madhesi ethnic community, as well as those with poor and less educated backgrounds to reduce the inequalities in maternal health care.

**Supplementary Information:**

The online version contains supplementary material available at 10.1186/s12884-023-05841-w.

## Background

Maternal health is the central pillar of a quality health system, as it is critical in reducing the number of deaths that occur during pregnancy and delivery [1]. The Sustainable Development Goal (SDG) 3 aims to reduce the global mortality ratio to less than 70 per 100,000 live births by 2030 [2]. Achieving this target requires maternal health services to address factors that either facilitate or impede the progress toward desirable outcomes, including low educational status, socio-economic deprivation, and geographic disadvantage. These factors can be compounded due to poor service quality [3], which can further exacerbate the challenges faced by women seeking maternal healthcare.

Antenatal care (ANC) is the first step in ensuring access to health services from pregnancy to childbirth and postpartum. Optimal ANC is essential for identifying and managing potential maternal complications during pregnancy, such as pre-eclampsia and gestational diabetes, a lack of coverage of which is detrimental for both mothers and their babies [4]. ANC also provides a valuable window of opportunity, to promote education regarding danger signs during pregnancy, birth spacing, and family planning.

The World Health Organization (WHO) recommended a minimum of four ANC visits during pregnancy until 2016. However, the WHO updated its guidelines and now recommends a minimum of eight ANC visits. Research has highlighted the importance of initiating ANC during the first three months of pregnancy (early initiation) and the uptake of recommended ANC components. Such components include having at least four ANC visits, consuming iron folic acid, deworming, and monitoring weight and blood pressure [1]. The components of ANC services can vary, depending on the service location due to the availability of laboratory facilities. For instance, it may be infeasible to monitor haemoglobin in rural and remote settings.

A national survey from Ghana reported that most mothers (80%) received four or more ANC visits [5] but only 55% commenced in the first trimester. Likewise, in India, 51.6% of mothers had 4 or more ANC visits [6], with the proportion of mothers consuming iron folic acid being only 30.8% [6], whereas it was 90.8% in Nigeria [7]. This variation demonstrates that ANC components can vary significantly across different settings. According to Nepal Demographic and Health Survey (NDHS) 2016, seven in ten women reported attending the recommended four or more ANC visits, and 91% said they had taken iron supplements [8].

Several studies have explored the coverage and quality of ANC visits in Nepal [1, 9, 10]. While most studies have focused on the number of ANC visits, one study did report on the timing of visits [10], and two have investigated the quality of ANC services [1, 11]. However, less is known about the intersection of socio-economic and geographic disadvantage and ANC services from the western terai and hill region of Nepal. This region has rich ethnocultural diversity, and is also marred by a higher incidence of poverty compared to other regions of Nepal, particularly among the Dalits and secluded groups. For example, the Nepal Living Standard Survey 2010/11 further analysis [12] showed that poverty incidence among Terai Dalits was four times higher than the hill Brahmins, a group regarded as socio-economically advantaged in Nepal.

Nepal has made tremendous progress in maternal health during the last two decades. The government has established policies and programs to increase access to ANC services by strengthening both structural and functional aspects and investing in human capital development. The government has been monitoring maternal, infant, and newborn mortality through national surveys [8] and studies [13] to make evidence-based decisions. Service utilization is also regularly monitored using the government’s national health management information system [14]. Despite these efforts, there are still under-utilization and poor quality of ANC in some regions, which recent studies have suggested can be addressed by tackling multiple social disadvantages [9, 15, 16]. A comprehensive knowledge of ANC and its inequitable service access is essential for the government to make an informed decision. Therefore, this study aimed to investigate the proportion of, and the factors associated with, key ANC services in western Nepal using social determinants of health framework whilst considering multiple markers of socio-economic deprivations [17].

## Methods

### Study setting

This study was conducted in the Rupandehi district of Nepal, which is situated in the western plain belt bordering India to the South. It is predominantly rural, with only two municipalities (urban areas) and 69 village development committees (rural areas) as the administrative units of the district. The largest hospitals and tertiary service outlets are located in urban areas. At the village level i.e. rural administrative unit, at least one government-managed primary health care centre/health post provides services. Although the district has relatively accessible roads, health and other services compared to hill and mountain regions of Nepal, it still takes many hours to reach the nearby hospital in many rural areas.

### Participants and procedure

A community-based prospective cohort study was conducted in January-October 2014. Only 18 mothers in urban areas declined to participate due to the possibility of migration, leaving a response rate of 97.6%. The mother-infant pairs were recruited within the first month of childbirth. Details of the recruitment and selection of participants had been reported previously [18–20]. Briefly, 15 rural and 12 urban locations were randomly selected from the Rupandehi district. A list of urban locations and rural VDC was available from the record of the local district public health office. A line listing exercise was done in the sampled areas to identify eligible mother-infant pairs with the help of local female community health volunteers and health facilities. The required sample of participants was selected randomly from the list.

The required number of mother-infant pairs per sample location was determined and selected in proportion to the population size based on the expected monthly infants aged < 30 days. The Ministry of Health and Population provides annual targets for health indicators, including the number of pregnant mothers and live births, among others, by location for the district public health office [18].

Face-to-face interviews were conducted by trained and experienced female enumerators recruited from the local communities. The research team provided them with additional training and practice sessions before data collection. The structured questionnaire was adapted from the Nepal Demographic and Health Survey and another similar study in the Kaski district of Nepal [21].

The mother-infant pairs were included and interviewed if they met the following criteria: the infant’s age was < 30 days, the mothers were residents of the community, single birth, and the child was alive at the time of recruitment [19]. The first author (VK) was deployed in the field throughout the study period to ensure the correct sampling process, train the local enumerators, and check the correctness and consistency of the information collected. The data from the baseline survey, i.e., the first 30 days of childbirth, were analyzed in this study.

#### Informed consent

was sought from each participant before enrolment and confidentiality of information collected was ensured. Mothers provided consent for themselves and their infants. All data were deidentified before statistical analysis.

### Variables

#### Outcome variables

This study investigated three key ANC outcomes: the number of ANC visits (categorized as underutilization if ≤ 3 visits and receiving adequate ANC if ≥ 4 visits). The timing of the ANC visit (categorized as late if a mother reported initiating at four months or after, or timely if they reported initiation at the first trimester) and the quality of ANC visits measured against nine components of the ANC visits (Table 1).

These two binary ANC outcomes on the number of ANC visits were aligned with the WHO and the Nepal Ministry of Health and Population guidelines [14]. A mother was included as ‘receiving ANC service’ irrespective of place of service delivery i.e., home (respondents’ home by village health workers or community workers, or health professional’s home), public or private health facility. The quality indicators were adapted from Joshi et al. [1]. In this study, the question concerning each component was read to the mothers who attended at least one ANC to solicit a binary ‘yes’ or ‘no’ response. We considered ‘optimal ANC’ was attained if a mother reported ‘yes’ to eight or more components and ‘suboptimal ANC’ if otherwise.

**Table 1:** Quality indicators of ANC care

Component	Item description	Response
1	Blood pressure measured	1 = Yes, 0 = No
2	Weight measured	1 = Yes, 0 = No
3	Iron-folic acid supplements given	1 = Yes, 0 = No
4	Deworming	1 = Yes, 0 = No
5	TT immunization	1 = Yes, 0 = No
6	Education on nutrition and hygiene	1 = Yes, 0 = No
7	Education on danger signs during pregnancy	1 = Yes, 0 = No
8	Education on danger signs during childbirth	1 = Yes, 0 = No
9	Identifying skilled birth attendant for childbirth	1 = Yes, 0 = No

#### Independent variables

Several independent variables were selected based on an extensive literature review. Maternal age was categorized as 15–19 years, 20–29 years, and 30–45 years which was originally recorded as a continuous variable. Maternal and partner education were categorized as no formal education, primary to lower secondary (year 1–8) and secondary and above (year 9 and above). Partner’s occupation was classified into employed (salaried job), semi-employed (labour, business and foreign labour), and unemployed (no paid job, unpaid subsistence agriculture) [20]. The study location encompassed multiple ethnic groups. Among them, Dalit is the most disadvantaged group. Nepal has a hierarchical caste-based system used by the government to classify “ethnicity”. The ethnic groups were classified into Dalits (Hill and Terai), Madhesi, Tharu (Indigenous group) and Hilly origin (non-Dalit). The place of residence was recorded as rural and urban. Regarding family wealth, a quintile variable was created based on the possession of key household assets (water source, toilet facility, types of cooking fuel, separate kitchen, floor material, electricity, radio, television, mobile phones, and cupboard). A principal component analysis was then applied to calculate a wealth score, which was further categorized into poor (lower 40%), middle (middle 40%) and rich (upper 20%) [18, 22]. Maternal smoking was recorded as either daily, sometimes, past smoker or non-smoker. Parity was dichotomized into primiparous and multiparous to facilitate analysis. Place of ANC visits were recorded as homes, hospitals, outreach clinics, health posts, private clinics, and nursing homes in the context of Nepal.

### Statistical analysis

Rates of under-utilization of ANC, late initiation of ANC visits, and sub-optimal ANC were reported using frequency distributions. The associations between these three outcomes and each independent variable were first examined using the Chi-square test. Multiple logistic regression analyses were then undertaken to ascertain their associations. All aforementioned independent variables that were significantly associated with outcome variables in Chi-square tests were initially entered into the model, followed by the backward stepwise selection procedure to determine pertinent significant factors. Adjusted Odds Ratios (AOR), together with their corresponding 95% confidence intervals (CI), were reported in the final models. A p-value of 0.05 was set as statistically significant. The data was entered and analyzed using Statistical Package for Social Science (SPSS) (IBM Corp, Armonk, NY, USA).

## Results

### Characteristics of the participants

A total of 735 mother-infant pairs participated in the observational study, with a response rate of 97.6%. Table 2 presents the characteristics of the mother-infant pairs who were recruited within 30 days of childbirth. The majority of the mothers were within the age bracket 20–29 years with mean of 24.6 years (SD: 4.6). About a quarter (26.1%) reported not attaining any formal education, whereas only 9.8% of their partners fell in the same educational attainment category. The majority of their partners (70.1%) were semi-employed, while 35.8% of participants belonged to the Madhesi ethnic group. Just over half (51.6%) of the participants came from rural areas, and 40% were from families in the poor wealth quintile. A vast majority of mothers (92.6%) reported as non-smokers. A total of 42.8% were primiparous, and only 6.6% received ANC service at home.

**Table 2:** Characteristics of participants and their association with ANC outcomes, Western Nepal

Characteristics	Distribution of sample (N = 735)	ANC Outcomes^#^		
	n (%)	Under-utilisation of ANC ( < = 3 ANC) visits	Late initiation of ANC	Sub-optimal ANC
Maternal age*		p = 0.059	P = 0.645	**p < 0.01**
15–19 years	66 (9.0)			
20–29 years	547 (74.8)			
30–45 years	118 (16.2)			
Maternal education*		**p < 0.001**	**p < 0.001**	**p < 0.001**
No formal education	191 (26.1)			
Primary to lower secondary	242 (33.1)			
Secondary and above	299 (40.8)			
**Partner’s education**		**p < 0.001**	**p = 0.005**	**p < 0.001**
No formal education	72 (9.8)			
Primary to lower secondary	305 (41.7)			
Secondary and above	355 (48.5)			
**Partner’s occupation**		P = 0.101	P = 0.508	P = 0.046
Employed	102 (13.9)			
Semi-employed	513 (70.1)			
Unemployed	117 (16.0)			
**Ethnicity**		**p = 0.01**	p = 0.220	**p < 0.001**
Dalit (Hill and Terai)	94 (12.8)			
Madhesi (non-dalit)	262 (35.8)			
Tharu	91 (12.4)			
Hilly origin (non-dalit)	285 (38.9)			
Type of family*		p = 0.96	p = 0.316	P = 0.026
Joint	528 (72.5)			
Nuclear	200 (27.5)			
**Place of residence**		p = 0.748	**p = 0.004**	p < 0.001
Rural	378 (51.6)			
Urban	354 (48.4)			
**Wealth status**		p = 0.069	**p < 0.001**	p < 0.001
Poor	294 (40.2)			
Middle	293 (40.0)			
Rich	145 (19.8)			
**Maternal smoking**		P = 0.671	P = 0.371	p < 0.001
Daily	26 (3.6)			
Sometimes or past smoker	28 (3.8)			
Non-smoker	678 (92.6)			
Parity*		**p < 0.001**	p = 0.136	**p = 0.028**
Primiparous	313 (42.8)			
Multiparous	418 (57.2)			
**Place of ANC service**		**p < 0.001**	**p = 0.009**	**p < 0.001**
Home	48 (6.6)			
Hospitals	268 (36.6)			
Outreach clinic	67 (9.2)			
Health posts	321 (43.9)			
Private clinics and nursing home	25 (3.8)			

P-value **Chi-square p-value; *missing value presents. ^**#**^ detailed results from cross tabulation provided in supplementary files

### Key ANC outcomes

**Fig. 1:**
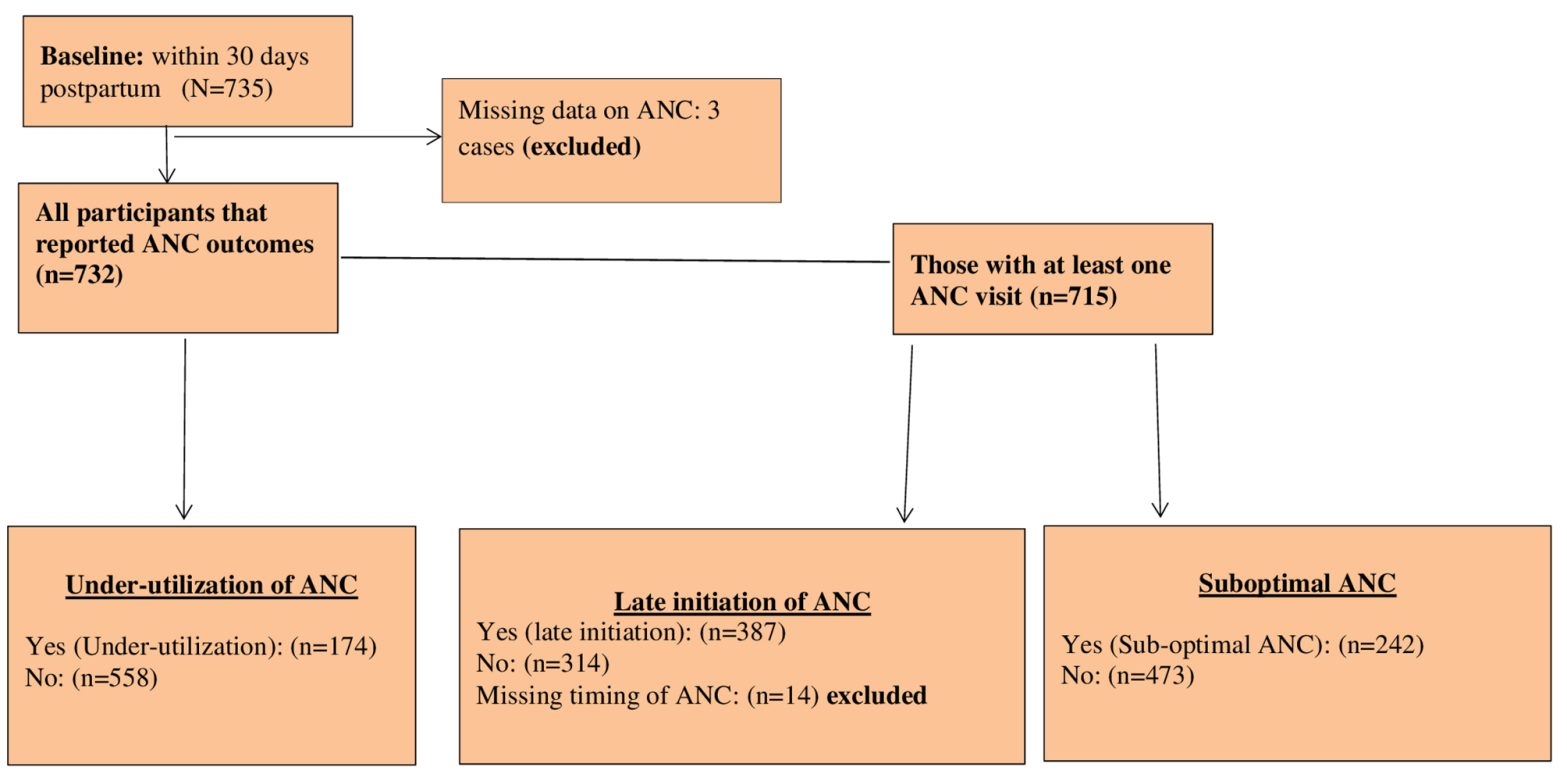
Flow chart of participants included for analysis

**Fig. 2:**
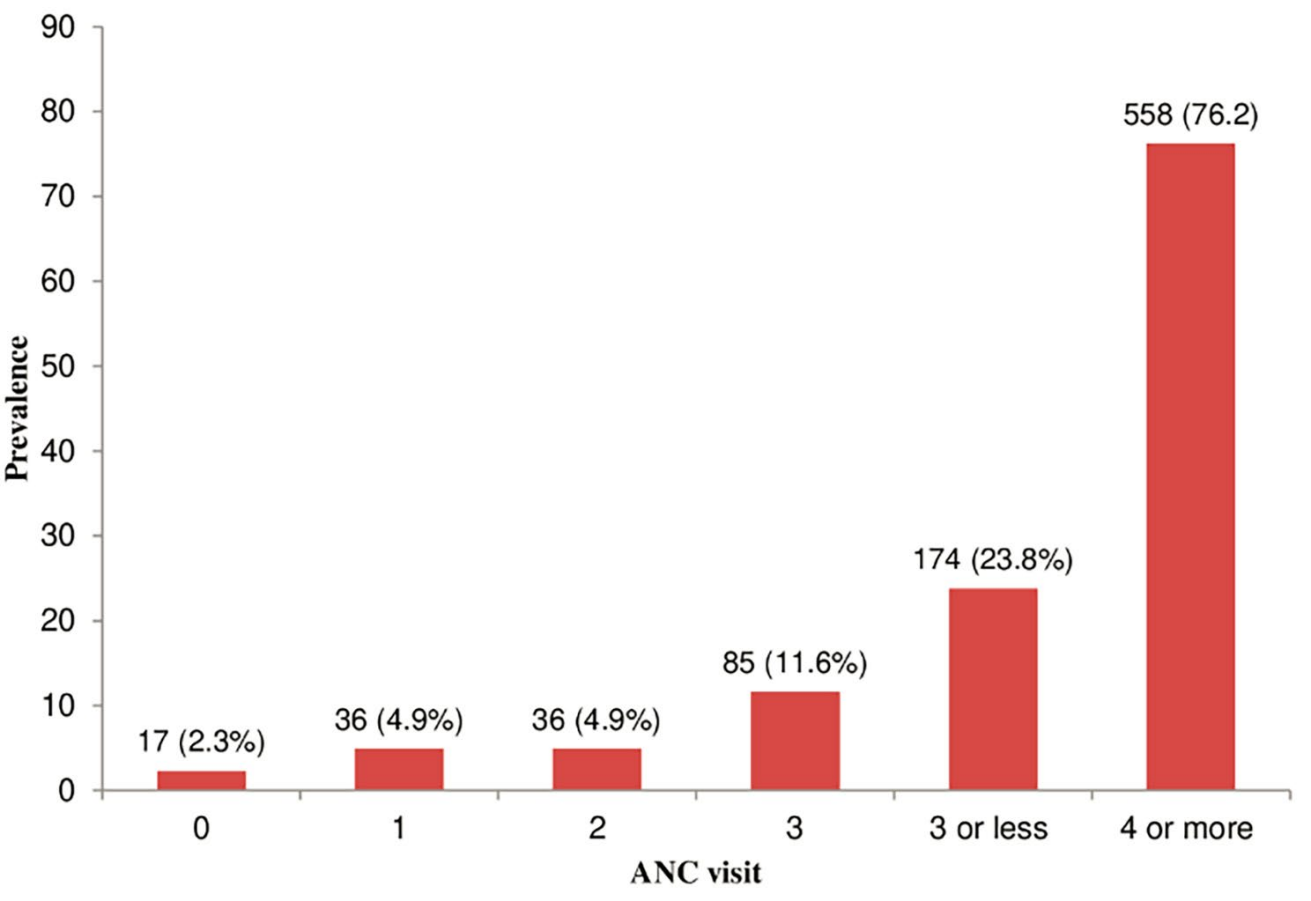
Distribution of antenatal care visits (N=732)

Figure 1 shows the flow chart of participants included for analyzing the three ANC outcomes of interest: under-utilization of ANC, late initiation of ANC and suboptimal ANC. Figure 2 presents the distribution of ANC visits. Only 2.3% of participants reported no ANC visit, while the observed rate of under-utilization was 23.8%.

Among the 715 mothers who attended at least one ANC, 701 reported their time of initiation. More than half of them, 387 (55.21%), reported late initiation of ANC (Fig. 1).

Concerning the quality of ANC, the high prevalence was observed for blood pressure measured (87.6%), weight measured (92.2%), iron-folic acid supplement given (95.2%), Albendazole (Deworming tablet) received (93.3%) and Tetanus toxoid immunization (91.3%). A lower proportion of them reported receiving the other four components: education on nutrition and hygiene (76.6%), education on danger signs during pregnancy (74.4%), education on danger signs during childbirth (70.1%) and identifying skilled birth attendants (68.8%). Overall, of the 715 mothers who paid at least one ANC visit, 242 (33.8%) received suboptimal ANC.

### Factors associated with ANC outcomes

Table 2 shows the results of factors significantly associated in cross-tabulation of the ANC outcomes against the independent variables. The detailed results of cross-tabulation are presented in supplementary Tables 1, 2, and 3. The significant correlates of ANC outcomes were further investigated in the regression models.

Table 3 presents the significant factors influencing the three ANC outcomes. Results from backward stepwise logistic regression analyses confirmed that mothers with no formal education (AOR: 2.02; 95% CI: 1.21, 3.37) and those with primary to lower secondary education (AOR: 1.99; 95% CI: 1.25, 3.17) were more likely to underutilize ANC services. Likewise, those who were multiparous (AOR: 1.13; 95% CI: 1.13, 2.47) had a higher likelihood of underutilization. On the other hand, women attending ANC at hospitals (AOR: 0.16; 95% CI: 0.08, 0.32), health posts (AOR: 0.20; 95% CI: 0.11, 0.38), and private clinics (AOR: 0.13; 95% CI: 0.03, 0.51) were less likely to underutilize when compared to others receiving ANC at home.

**Table 3:** Factors influencing ANC outcomes in Western Nepal

Factors	Underutilisation of ANC AOR (95% CI).	Late initiation of ANC AOR (95% CI).	Suboptimal ANC AOR (95% CI).
**Maternal Education**			
Secondary and above	1.00	1.0	1.0
No formal education	2.02 (1.21, 3.37)*	2.14 (1.34, 3.43)*	2.7 (1.60, 4.66)*
Primary to lower secondary	1.99 (1.25, 3.17)*	2.08 (1.39, 3.13)*	1.67 (1.05, 2.66)*
**Parity**			
Primiparous	1.00		
Multiparous	1.17 (1.13, 2.47)*		
**Place of service delivery**			
Home	1.00	1.0	
Hospitals	0.16 (0.08, 0.32)*	0.49 (0.21, 1.12)	
Outreach clinic	0.19 (0.09, 0.46)*	0.42 (0.16, 1.10)	
Health posts	0.20 (0.11, 0.38)*	0.67 (0.29, 1.56)	
Private clinics and nursing home	0.13 (0.03, 0.51)*	0.27 (0.08, 0.87)*	
**Place of residence**			
Rural		1.0	1.0
Urban		3.06 (2.03, 4.60)*	1.49 (1.01, 2.22)*
**Wealth** status			
Rich		1.0	
Poor		2.32 (1.43, 3.78) *	
Middle		1.42 (0.92, 2.19)	
**Maternal age**			
15–19 years			1.0
20–29 years			0.47 (0.27, 0.83)*
30–45 years			0.81 (0.41, 1.59)
**Maternal smoking**			
Non-smoker			1.0
Daily			2.59 (1.05, 6.37)*
Sometimes or past smoker			1.09 (0.46, 2.58)
**Ethnicity**			
Dalit (Hill and Terai)			1.0
Madhesi (non-dalit)			2.63 (1.49, 4.61)*
Tharu			1.21 (0.60, 2.44)
Hilly origin (non-dalit)			1.70 (0.89, 3.25)

*AOR: Adjusted Odds Ratio. 95%CI: 95% Confidence* Interval. *p-value < 0.05

Regarding timely initiation of ANC, mothers with no formal education (AOR: 2.14; 95% CI: 1.34, 3.43), primary to lower secondary education (AOR: 2.08; 95% CI: 1.39, 3.13), residing in rural areas (AOR: 3.06; 95% CI: 2.03, 4.60) and with poor family wealth background (AOR: 2.32; 95% CI: 1.43, 3.78), were more likely to initiate ANC late. On the other hand, mothers who went to private clinics for their ANC services were less susceptible to late initiation (AOR: 0.27; 95% CI: 0.08, 0.87).

Regarding the quality of ANC, mothers with no formal education (AOR: 2.73; 95% CI: 1.60, 4.66), primary to lower secondary education (AOR: 1.67; 95% CI: 1.05, 2.66), from the Madhesi ethnic group (AOR: 2.63; 95% CI: 1.49, 4.61), residing in rural areas (AOR: 1.49; 95% CI: 1.01, 2.22) and reported smoking daily (AOR: 2.59; 95% CI: 1.05, 6.37), were more likely to receive suboptimal ANC. On the contrary, mothers aged 20–29 years were at lower risk (AOR: 0.47; 95% CI: 0.27, 0.83) of receiving sub-optimal ANC than their younger counterparts.

## Discussion

Adequate, timely, and quality ANC utilization are measures that have been used widely to evaluate maternal health [23]. This study investigated the inequalities in access to quality ANC services, the recommended number of visits, the time of initiation of ANC visits, and the quality of ANC services received by mothers in the western region of Nepal. Although only 2.3% of participants reported no ANC visit and over three-quarters attended four or more visits, more than half of the women were unable to initiate ANC as recommended and not all recommended services were offered when they did start ANC. This suggests that despite a significant achievement in ANC coverage, gaps in the quality of the service persist. Maternal education, place of residence, ethnicity, and family wealth appeared as the key social determinants of health affecting access to and quality of ANC services.

### Underutilization of ANC

The majority of women (76%) in this study attended four or more ANC visits; however, it varied according to their education status, parity, and place of service delivery. The study identified maternal education as a strong indicator of ANC service utilization. Previous studies conducted in Nepal, Ethiopia, and Burundi found that women with secondary education or above have higher odds of receiving the recommended four ANC visits compared to women with no education [24, 25]. Women with a low level of education may not be aware of the importance of prenatal care [26]. We also observed an inverse association between pregnancy order and utilization of ANC service. The multiparous women appeared to be less likely to attain the recommended minimum of four ANC visits is consistent with previous studies carried out in low- and middle-income countries [1, 27, 28]. Women with birth experience might not be interested in attending ANC due to their greater confidence arising from previous pregnancies. Moreover, our study demonstrated a significant difference in ANC utilization by place of service delivery and was consistent with another study conducted in India [29], which found that ANC utilization was low for home-based service.

### Late initiation of ANC

In this study, less than half of the participants (45%) initiated their ANC visit within the first trimester of pregnancy. A recent systematic review using a demographic health survey and multiple cluster indicator survey of 54 low- and middle-income countries reported 44.3% of all women had timely ANC initiation [23]. However, the early initiation rate was lower than the 70% found in other regions of Nepal [10]. This partly explains the barrier to early access to and continuity of care in maternal health.

Our study found the place of residence was associated with the timely initiation of ANC, suggesting the vulnerability of women in rural areas. Their delayed initiation is consistent with findings from previous studies [23, 27, 30]. It might be due to issues related to the availability, accessibility, and affordability of healthcare services in rural areas.

Maternal education was also associated with the initiation of ANC. The less educated mothers appeared to be reluctant to commence ANC on time. This finding is similar to other observations in Nepal, Tanzania, Nigeria, and other 54 low and middle-income countries [10, 23, 31]. It is logical that educated women are more aware of the importance of timely initiation of ANC, and also resonates with the role of education in relation to the Sustainable Development Goal (SDG) 4: improve quality education for all girls and boys by 2030 showing multifaceted benefits of the education [2].

Household wealth was found to be inversely associated with the late initiation of ANC. Previous studies have reported similar findings [10, 23]. In our sample, only a small proportion of mothers from poor families went to the private sector for ANC.

### Suboptimal ANC

About two-thirds of the participants received quality ANC services. This rate was higher than previous reports in other regions of Nepal [1, 16]. While this is encouraging, there are still many mothers who reported receiving suboptimal quality ANC.

Maternal education was positively associated with receiving quality ANC service, where women without formal education were more likely to receive suboptimal ANC than their counterparts with higher education. Previous studies have already highlighted the relationship between maternal education and quality of ANC services [1, 5].

Alhough the ethnicity of women was associated with the quality of ANC, previously conducted studies in Nepal and India did not report such an association between the quality of ANC and disadvantaged ethnicity [16]. It should be noted that these studies adopted different combined ethnicity groups, so a direct comparison might not be feasible. Nevertheless, our result showed that women from the Madhesi ethnic group were vulnerable and warranted attention when planning further maternal health programs.

Similar to the previous studies [1, 5], our study revealed that pregnant women from rural areas were more likely to receive suboptimal ANC services. Less equipped health facilities, poor staffing, difficult access to the facilities, and inadequate staff capacity can be the underlying reasons [32].

Adolescent pregnant women were less likely to receive quality ANC services than their older counterparts, consistent with other studies [29]. Being married and pregnant in young age limits their ability to complete their education and get employment.

In this study, the smoking habit of pregnant women was associated with the quality ANC service. Smokers appeared to be more vulnerable to receiving suboptimal ANC. It is a new finding that has not been identified in the literature, and further studies may explore the multiple health and social disadvantages they face.

### Strengths and limitations

The present study was the first community-based study on ANC inequalities conducted in western Nepal that adopted a short recall period of 30 days postpartum. This study also has an adequate sample size and used the trained enumerator to minimize errors during the data collection. The first author was extensively engaged in monitoring data collection, checking, and verification of the information collected. The self-report of ANC timing and quality could be affected by recall error; however, it is still recognized as the most acceptable method to document maternal health service utilization in resource-poor settings. Since the participants were prompted to respond to each component of ANC, their reported prevalence should reflect the reality at the community level. Another limitation of the study is residual confounders. While we attempted to include as many variables as possible based on the literature review, it is possible that there are other variables that are not part of the study and would affect the ANC outcomes. Our findings also call for a higher focus on girl’s education (SDG 4) in achieving ‘Ensure healthy lives and promote well-being for all at all ages’ (SDG 3) [2]. Since this study was conducted, the Ministry of Health and Population in Nepal now recommends eight or more ANC visits as optimum. Future studies need to consider such changes in the recommendations, and caution should be taken while comparing the findings.

## Conclusions

This study found that about three-quarters of mothers in western Nepal attended the recommended four and more ANC visits. However, only half of them commenced their visits on a timely basis, and one-third reported receiving suboptimal ANC. Maternal education remained a consistent factor affecting ANC service utilization. Likewise, women residing in rural areas and those from the Madhesi ethnic group were more likely to have received suboptimal quality of ANC. Therefore, future health interventions to improve access and quality need to target these vulnerable groups given the apparent inequalities.

### Electronic supplementary material

Below is the link to the electronic supplementary material.

Supplementary Material 1

## Data Availability

The project data is kept under the data protection regulation of Curtin University, Australia and can be obtained with the reasonable request from VK and AHL.

## List of abbreviations

ANC: Antenatal Care
SDG: Sustainable Development Goals
AOR: Adjusted Odds Ratio
CI: Confidence Interval
WHO: World Health Organisation
