# Supplementary material for: Dissecting antenatal care inequalities in western Nepal: insights from a community-based cohort study
Source: BMC Pregnancy Childbirth. 2023 Jul 17;23:521. doi: 10.1186/s12884-023-05841-w (PMC10353079; doi:10.1186/s12884-023-05841-w)
Supplement: Supplementary file 1 — Supplementary Material 1 [file 12884_2023_5841_MOESM1_ESM.docx]

**Supplementary Table 1 Utilisation of ANC by participant characteristics of participants, western Nepal (N=732)**

| **Characteristics** | **Utilization of ANC Visits** | | **P-value** |
| --- | --- | --- | --- |
|  | **≥ 4 ANC**  **n (%)** | **Under-utilisation (≤ 3) n (%)** |  |
| **Maternal age*** |  |  | P=0.059 |
| 15-19 years | 50 (75.8) | 16 (24.2) |  |
| 20-29 years | 427(78.1) | 120 (21.9) |  |
| 30-45 years | 80(67.8) | 38 (32.2) |  |
| Maternal education |  |  | **P<0.001** |
| No formal education | 131 (68.6) | 60 (31.4) |  |
| Primary to lower secondary | 170 (70.2) | 72 (29.8) |  |
| Secondary and above | 257 (86.0) | 42 (14.0) |  |
| **Partner’s education** |  |  | **P<0.001** |
| No formal education | 44 (61.1) | 28 (38.9) |  |
| Primary to lower secondary | 217 (71.1) | 88 (28.9) |  |
| Secondary and above | 297 (83.7) | 58 (16.3) |  |
| **Partner’s occupation** |  |  | P=0.101 |
| Employed | 86 (84.3) | 16 (15.7) |  |
| Semi-employed | 382 (74.5) | 131 (25.5) |  |
| Unemployed | 90 (76.9) | 27 (23.1) |  |
| **Ethnicity** |  |  | **P=0.01** |
| Dalit (Hill and Terai) | 66 (70.2) | 28 (29.8) |  |
| Madhesi (non-dalit) | 183 () | 73 () |  |
| Tharu | 67 (72.1) | 24 (27.9) |  |
| Hilly origin (non-dalit) | 236 (82.8) | 49 (17.2) |  |
| **Type of family*** |  |  | P=0.96 |
| Joint | 403 (76.3) | 125 (23.7) |  |
| Nuclear | 153 76.5() | 47 (23.5) |  |
| **Place of residence** |  |  | P=0.748 |
| Rural | 290 (76.7) | 88 (23.3) |  |
| Urban | 268 (75.7) | 86 (24.3) |  |
| **Wealth** status |  |  | P=0.069 |
| Poor | 214 (72.8) | 80 (27.2) |  |
| Middle | 224 (76.5) | 69 (23.5) |  |
| Rich | 120 (82.8) | 25 (17.2) |  |
| **Maternal smoking** |  |  | P=0.671 |
| Daily | 18 (69.2) | 8 (30.8) |  |
| Sometimes or past smoker | 22 (78.6) | 6 (21.4) |  |
| Non-smoker | 518 (76.4) | 160 (23.6) |  |
| **Parity*** |  | **P<0.001** |  |
| Primiparous | 260 (83.1) | 53 (16.9) |  |
| Multiparous | 297 (71.1) | 121 (28.9) |  |
| **Place of ANC service** |  |  | **P<0.001** |
| Home | 18 (37.5) | 30 (62.5) |  |
| Hospitals | 223 (83.2) | 45 (16.8) |  |
| Outreach clinic | 49 (73.1) | 18 (26.9) |  |
| Health posts | 243 (75.7) | 78 (24.3) |  |
| Private clinics and nursing home | 25 (89.3) | 3 (10.7) |  |

P-value = chi-square p-value; *=missing value presents.

**Supplementary Table 2 Initiation of ANC visits by participants characteristics, western Nepal (N=701)**

| **Characteristics** | **Time of initiation of ANC** | | **P value** |
| --- | --- | --- | --- |
|  | **Timely initiation (314)**  **n (%)** | **Late initiation (387)**  **n (%)** |  |
| **Maternal age*** |  |  | P=0.645 |
| 15-19 years | 32 (50.0) | 32 (50.0) |  |
| 20-29 years | 232 (44.0) | 295 (56.0) |  |
| 30-45 years | 50 (45.9) | 59 (54.1) |  |
| Maternal education |  |  | **P<0.001** |
| No formal education | 69 (38.5) | 110 (61.5) |  |
| Primary to lower secondary | 86 (37.4) | 144 (62.6) |  |
| Secondary and above | 159 (54.5) | 133 (45.5) |  |
| **Partner’s education** |  |  | **P=0.005** |
| No formal education | 27 (40.9) | 39 (59.1) |  |
| Primary to lower secondary | 110 (38.3) | 177 (61.7) |  |
| Secondary and above | 177 (50.9) | 171 (49.1) |  |
| **Paternal occupation** |  |  | P=0.508 |
| Employed | 51 (50.0) | 51 (50.0) |  |
| Semi-employed | 217 (44.1) | 275 (55.9) |  |
| Unemployed | 46 (43.0) | 61 (57.0) |  |
| **Ethnicity** |  |  | P=0.220 |
| Dalit (Hill and Terai) | 31 (36.5) | 54 (63.5) |  |
| Madhesi (non-dalit) | 107 (43.1) | 141 (56.9) |  |
| Tharu | 40 (44.9) | 49 (55.1) |  |
| Hilly origin (non-dalit) | 136 (48.7) | 143 (51.3) |  |
| **Type of family*** |  |  | P=0.316 |
| Joint | 235 (45.9) | 277 (54.1) |  |
| Nuclear | 77 (41.6) | 108 (58.4) |  |
| **Place of residence** |  |  | **P=0.004** |
| Rural | 182 (50.0) | 182 (50.0) |  |
| Urban | 132 (39.2) | 205 (60.8) |  |
| **Wealth** status |  |  | **P<0.001** |
| Poor | 102 (35.7) | 184 (64.3) |  |
| Middle | 138 (48.6) | 146 (51.4) |  |
| Rich | 74 (56.5) | 57 (43.5) |  |
| **Maternal smoking** |  |  | P=0.371 |
| Daily | 14 (53.8) | 12 (46.2) |  |
| Sometimes or past smoker | 15 (55.6) | 12 (44.4) |  |
| Non-smoker | 285 (44.0) | 363 (56.0) |  |
| **Parity*** |  |  | P=0.136 |
| Primiparous | 147 (47.9) | 160 (52.1) |  |
| Multiparous | 166 (42.2) | 227 (57.8) |  |
| **Place of ANC service delivery** |  |  | **P=0.009** |
| Home | 9 (29.0) | 22 (71.0) |  |
| Hospitals | 121(46.5) | 139 (53.5) |  |
| Outreach clinic | 35 (53.8) | 30 (46.2) |  |
| Health posts | 130 (41.0) | 187 (59.0) |  |
| Private clinics and nursing home | 19 (67.9) | 9 (32.1) |  |

P-value = chi-square p-value; *=missing value presents.

**Supplementary Table 3 Quality of antenatal visits by participants characteristics, western Nepal (N=715)**

| **Characteristics** | **Quality of ANC** | | **P-value** |
| --- | --- | --- | --- |
|  | **Optimal ANC (n=473)**  **n (%)** | **Sub-optimal ANC (n=242)**  **n (%)** |  |
| **Maternal age*** |  |  | **<0.01** |
| 15-19 years | 37 (56.9) | 28 (43.1) |  |
| 20-29 years | 377 (70.5) | 158(29.5) |  |
| 30-45 years | 59 (51.8) | 55 (48.2) |  |
| Maternal education |  |  | **<0.001** |
| No formal education | 90 (48.4) | 96 (51.6) |  |
| Primary to lower secondary | 147 (63.4) | 85 (36.6) |  |
| Secondary and above | 236 (79.5) | 61(20.5) |  |
| **Partner’s education** |  |  | **<0.001** |
| No formal education | 27 (39.7) | 41(60.3) |  |
| Primary to lower secondary | 186 (62.8) | 110 (37.2) |  |
| Secondary and above | 260 (74.1) | 91 (25.9) |  |
| **Partner’s occupation** |  |  | 0.046 |
| Employed | 77 (75.5) | 25 (24.5) |  |
| Semi-employed | 328 (65.7) | 171(34.3) |  |
| Unemployed | 68 (59.6) | 46 (40.4) |  |
| **Ethnicity** |  |  | **<0.001** |
| Dalit (Hill and Terai) | 65 (73.0) | 24 (27.0) |  |
| Madhesi (non-dalit) | 125 (49.2) | 129 (50.8) |  |
| Tharu | 65 (72.2) | 25 (27.8) |  |
| Hilly origin (non-dalit) | 218 (77.3) | 64 (22.7) |  |
| **Type of family*** |  |  | 0.026 |
| Joint | 332 (63.8) | 188 (36.2) |  |
| Nuclear | 139 (72.8) | 52 (27.2) |  |
| **Place of residence** |  |  |  |
| Rural | 218 (58.1) | 157 (41.9) |  |
| Urban | 255 (75.0) | 85 (25.0) |  |
| **Wealth** status |  |  | <0.001 |
| Poor | 167 (58.0) | 121 (42.0) |  |
| Middle | 205 (71.7) | 81 (28.3) |  |
| Rich | 101 (71.6) | 40 (28.4) |  |
| **Maternal smoking** |  |  | <0.001 |
| Daily | 8 (30.8) | 18 (69.2) |  |
| Sometimes or past smoker | 19 (67.9) | 9 (32.1) |  |
| Non-smoker | 446 (67.5) | 215 (32.5) |  |
| **Parity*** |  |  | **0.028** |
| Primiparous | 220 (70.5) | 92 (29.5) |  |
| Multiparous | 252 (62.7) | 150 (37.3) |  |
| **Place of ANC service delivery** |  |  | **<0.001** |
| Home | 19 (61.3) | 12 (38.7) |  |
| Hospitals | 200 (74.6) | 68 (25.4) |  |
| Outreach clinic | 26 (38.8) | 41 (61.2) |  |
| Health posts | 206 (64.2) | 115 (35.8) |  |
| Private clinics and nursing home | 22 (78.6) | 6 (21.4) |  |

P-value = chi-square p-value; *=missing value presents.
